# Notes and News

**Published:** 1900-03-10

**Authors:** 


### Notes and News.

### HOSPITAL MEETINGS.

Hospital for Diseases of the Skin, Stamford Street, S.E.—Annual Meeting at five p.m., on Monday, March 12th, at the Hospital.

West Ham Hospital, Stratford, E.—Annual Meeting at eight p.m., on Wednesday, March 14th.

Royal Hospital for Diseases of the Chest, City Road.—Annual Meeting at three p.m, on Thursday, March 15th, at the Hospital.

Dental Hospital of London, Leicester Square.—Annual General Meeting at half past five p.m., on Thursday, March 15th, at the Hospital.

New Hospital for Women, Euston Road.—Annual Meeting at half-past three p.m., on Wednesday, March 14th.

The committee of the Liverpool School of Tropical Diseases will shortly send out another expedition for the investigation of malaria to the West Coast of Africa.

The plague is still on the increase in India. In Bombay the mortality is very high. Small-pox has also been exceptionally prevalent, whilst cholera and dysentery are much less than usual.

Mr. Robert Edwin Matheson has been appointed Registrar-General for Ireland, in place of the late Dr. Grimshaw. Mr. Matheson is a Member of the Bar, and has been secretary at the office of the Registrar-General for many years. He has done some valuable work in connexion with the census.

The lengthy correspondence which has passed between the authorities of St. Mark's Hospital and the Prince of Wales's Fund, the commencement of which we republished from the *Times* in our issue of February 24th, and a further instalment of which will be found in another column of the present issue, has caused us to look into the reports of the St. Mark's Hospital. From these it appears that the festival dinner produced a profit of £2,720, not merely £1,720 as mentioned, of which £1,000 was placed upon deposit, and the balance transferred to the current income and expenditure account, as is usual and proper. In addition to this, we find that St. Mark's Hospital has invested funds amounting to upwards of £20,000, a sum exceeding five years' ordinary expenditure, so that, as a matter of fact, it is not only incorrect, but in our judgment improper, to talk of a hospital possessing this financial strength as "urgently in need of funds," or "sorely in need of increased assistance."

Sir William MacCormac left Cape Town for England on Saturday.

Another naval hospital is to be established at Sheerness. The estimated cost is £12,000.

The Italian Ambassador will open the new buildings of the Italian Hospital, Queen Square, on Wednesday, March 14th.

A case of bubonic plague is reported as having arrived on a transport at Cape Town from Rosario and Buenos Ayres.

The Princess of Wales has consented to become the patroness of the Davos Invalids' Home, which was founded in 1884 by the late Mrs. Lord.

The magnificent new infirmary for Bethnal Green has been opened by Mr. W. J. Webster, chairman of the Board of Guardians. The site cost £36,000, and the equipped and furnished building £175,000. The accommodation is for 669 patients.

The Barry District Nursing Association and the Accident Hospital have been disconnected. The Accident Hospital has now been taken over by the Barry Urban District Council, and will be the first hospital supported entirely out of the rates.

Poor Ladysmith has gone through a hotter time than some of us seem to have imagined. The cheerful heliograph kept up our spirits; but now when we find 800 cases of typhoid fever in the town we recognise how hard pressed the heroic defenders really were—not by Boers, but by starvation and disease.

An interesting competition has been organised by the Italian Government in connection with the Tuberculosis Congress at Naples in April. Two prizes of £200 and £120 respectively are offered for the first and second best plans of a sanitorium for consumption. The competition is announced as open to Italians only.

The United States authorities are taking great care to prevent the introduction of the plague into the City. Fifteen officers of the United States Marine Hospital Service have been stationed at the same number of foreign ports, to report as to the health of the vessels and of the various cities at which they are stationed.

A deputation representing the medical hospitals and infirmaries of Great Britain and the Central Hospital Council for London has been received by Mr. Chaplin to plead for the exemption of voluntary public hospitals from the payment of rates. These institutions are maintained for the public good, at a cost of £1,500,000 a year.

Through the International Institute of Scientific Bibliography in Paris the first number of *Bibliographica Medica* has been issued. This publication is on the lines of the American *Index Medicus*, conducted until lately by Billings and Flechter. It employs, however, a different classification. It will deal with the whole of International medical bibliography, and it will be issued monthly, each number containing about eighty pages. The price for foreign countries is 60 francs. Dr. C. Potani and Dr. Charles Richet are the directors.

